# A cross-species judgement bias task: integrating active trial initiation into a spatial Go/No-go task

**DOI:** 10.1038/s41598-018-23459-3

**Published:** 2018-03-23

**Authors:** Sara Hintze, Luca Melotti, Simona Colosio, Jeremy D. Bailoo, Maria Boada-Saña, Hanno Würbel, Eimear Murphy

**Affiliations:** 1 Division of Animal Welfare, University of Bern, Länggassstrasse 120, 3012 Bern, Switzerland; 2 Division of Livestock Sciences, Department of Sustainable Agricultural Systems, University of Natural Resources and Life Sciences Vienna (BOKU), Gregor-Mendel-Strasse 33, 1180 Vienna, Austria; 3 RG Behavioural Biology & Animal Welfare, Division of Behavioural Biology, University of Münster, Badestrasse 13, 48149 Münster, Germany

## Abstract

Judgement bias tasks are promising tools to assess emotional valence in animals, however current designs are often time-consuming and lack aspects of validity. This study aimed to establish an improved design that addresses these issues and can be used across species. Horses, rats, and mice were trained on a spatial Go/No-go task where animals could initiate each trial. The location of an open goal-box, at either end of a row of five goal-boxes, signalled either reward (positive trial) or non-reward (negative trial). Animals first learned to approach the goal-box in positive trials (Go) and to re-initiate/not approach in negative trials (No-go). Animals were then tested for responses to ambiguous trials where goal-boxes at intermediate locations were opened. The Go:No-go response ratio was used as a measure of judgement bias. Most animals quickly learned the Go/No-go discrimination and performed trials at a high rate compared to previous studies. Subjects of all species reliably discriminated between reference cues and ambiguous cues, demonstrating a monotonic graded response across the different cue locations, with no evidence of learning about the outcome of ambiguous trials. This novel test protocol is an important step towards a practical task for comparative studies on judgement biases in animals.

## Introduction

Measuring emotional states in animals is a critical goal for the assessment of animal welfare, e.g.^1^. Most researchers agree that the subjective experience of emotion cannot be assessed directly in other animals (but see e.g.^2,3^ for alternative approaches). However, emotions are multifaceted states that besides the individual´s subjective experience also include behavioural, physiological and cognitive components^4^, all of which can be assessed objectively. Thus, the true challenge facing animal welfare scientists may be to identify objective indicators of emotions, and reliable and valid methods to assess them^5^.

Of particular interest to animal welfare science are methods for the assessment of emotional valence, the positivity or negativity of emotional states as perceived by the animal. One of the most promising approaches to the study of emotional valence is the use of cognitive biases as proxy measures^6^. In particular, as originally proposed by Harding and colleagues^7^, cognitive judgement biases in response to ambiguous stimuli may allow us to assess variation in emotional valence. Thus, optimistic judgements of ambiguous stimuli are considered to reflect underlying positive emotional states, whereas pessimistic judgements are considered to reflect underlying negative emotional states, e.g.^7,8^. Tasks to assess judgement biases generally proceed across two stages^7^: In the first stage, the animals are trained to discriminate two reference cues predicting a positive and a negative outcome; in the second stage, they are presented with intermediate and thus ambiguous cues interspersed among the reference cues. The ratio of optimistic (behavioural response indicating expectation of positive outcome) to pessimistic (behavioural response indicating expectation of negative outcome) responses to ambiguous cues is interpreted in terms of positive and negative emotional valence. Even though the basic principle has remained the same, a wide variety of cue modalities (e.g. auditory, spatial, olfactory, tactile, and visual cues), outcome measures (e.g. latency-based tasks, choice-based tasks), and task designs have been applied, e.g.^8–11^, but no study has aimed to apply the same task protocol across species. In fact, a study using a counterbalanced Go/No-go task (i.e. Go for positive cue, No-go for negative cue vs. Go for negative cue, No-go for positive cue) for laboratory rodents using a shuttle box, found that mice and rats learned opposite, but not both, contingencies^12^.

With the implementation of different task designs and further research into the underlying assumptions of these tasks, a number of practical and theoretical limitations have become apparent that may question the practicality of tasks for testing judgement biases on a wider scale, e.g.^11^, as well as the validity of inferences in terms of emotional valence. For example, the initial reference cue discrimination training may be very time consuming due to long trial times (e.g. 2 min^13^ and 90 s^14^), which in sum contribute to lengthy training sessions and a large number of sessions to reach criterion, e.g. up to 53 sessions^15^. Such prolonged training could in itself influence the animals’ responses to ambiguous cues, thereby potentially masking or confounding treatment effects^16^. In addition, judgement bias tasks are based on several assumptions^17^, not all of which are generally met.

In particular, to ensure that the ambiguous cues are evaluated within the context of the task (i.e. in relation to the reference cues), and not simply as novel or irrelevant^8^, animals should show a monotonic graded response across the ambiguous cues (a response curve which is neither flat nor erratic^8,12,17^ but maintains the same direction, i.e. increasing optimistic responses across the ambiguous cues the closer an ambiguous cue is to the positive cue). Such a slope indicates that the ambiguous cues are interpreted with reference to the previously learned positive and negative cues – a requirement to demonstrate the internal validity of the task, which has been violated in several studies, e.g.^15,18,19^, and without which treatment differences may not reflect differences in emotional valence.

Finally, judgement bias tasks depend on the repeated presentation of ambiguous cues. If the animals can learn about the outcomes associated with ambiguous cues, these cues, by definition, are no longer ambiguous^9,11^. Various methods have been proposed to reduce the probability of learning about the outcomes of ambiguous cues both at the stage of training, e.g. variable reinforcement ratios^20^/partial reinforcement^21^, and at the stage of testing, e.g. by not rewarding Go responses to ambiguous cues, e.g.^18^, or rewarding according to expectation, e.g.^22^. However, no systematic research has been conducted on how best to do this.

Based on these general issues, the aim of the present research was to develop a judgement bias task that can be used across different species of animals, is relatively quick to learn, and meets important assumptions of judgement bias tasks in terms of the pattern of responses and the repeatability of ambiguous cue presentations. The general task design and test protocol were developed based on the available literature, previous experience of the authors with judgement bias tasks in horses, rats, mice and pigs, and pilot tests to assess the practicability of different aspects of the task design and test protocol. The final protocol was based on a spatial Go/No-go task, whereby the location of an open goal-box within a series of goal-boxes adjacent to each other signalled either the presence or absence of a food reward. Spatial discrimination was chosen because it is one of the most commonly used paradigms to assess cognition in captive animals, and has been successfully applied in a number of judgement bias task designs in different species. The spatial cue (the open goal-box) was emphasised using both auditory and visual signals which were features of the spatial cue itself (requiring minimal additional association learning) and thus maximised the salience of the spatial cue. In addition, using an operant response (trial initiator), the animals were given the opportunity to skip waiting time when opting for a No-go response and proceed immediately to the next trial. The trial initiator provides the animals with a higher degree of control over the task, compared to previous task designs, which should help reduce frustration in negative trials and keep the animals focused on the task (e.g. less off-task behaviour). Further, perceived control over the task could keep the animals motivated to perform as it has been demonstrated in other species^23^, thereby maximising the number of trials per training session and the overall training speed. The present study used the final protocol and assessed its applicability to different species of animals. To this end, we used horses, laboratory rats, and laboratory mice to cover a range of mammals differing in body size, sensory capacities, and handling levels (lower for lab rodents and higher for horses).

If our task is successful, we hypothesise that (1) it can be learned across species and strains (horses, rats, two strains of mice), (2) learning should be relatively quick both in regards of number of sessions and session length, (3) animals should show a monotonic graded response towards the ambiguous cues when tested, as proposed by Gygax^17^, both at the group and at the individual level, and (4) due to the relatively high number of positive and negative trials per session, a large number of ambiguous cues can be presented without the animals learning the association between trial type and outcome.

## Material and Methods

Each species was trained and tested independently on the Judgement Bias Task (JBT) by different experimenters. However, a similar training and test protocol was used, with only small modifications to account for species-specific differences (see Supplementary Information). Data were analysed independently for all species and strains but all analyses were run in the same way by the same experimenter to guarantee comparability between them.

### Animals and housing

An overview of the different animal species and strains is given in Table 1.

**Table 1:** Overview of the animals of all three species and the two mice strains used in the study.

Species	Strain/Breed	Sex	Origin (Rodents) Location (Horses)	Batch	Sample size	Age at start of study
Horses	Franches-Montagnes	♂	Swiss National Stud Farm	1 2	12 13	4–14 years 5–22 years
Rats	Lister Hooded	♂	Charles River Laboratories	1 2	8 8	8.5 months 9 month
Mice	C57BL6/JRj (C57)	♀	Janvier Labs	1 2	12 12	3.5 months 3.5 months
Mice	RjOrl:SWISS (SWISS)	♀	Janvier Labs	1 2	12 12	3.5 months 3.5 months

### Horses

In total 25 Franches-Montages stallions, housed at the Swiss National Stud Farm of Agroscope, Avenches, Switzerland, were trained on the JBT in two batches of 12 and 13 horses, respectively. All horses were part of a larger study on fearfulness^24^, half of which were selected from those classified as high fearful and the other half was selected from those classified as low fearful. Because of the need for further validation of the fearfulness measures taken in the larger study, and since the aim of the current study was to assess the internal validity of the JBT across species, the effect of fearfulness on judgement bias in the horses was not investigated in this study. Horses were housed individually in standard single boxes (3 × 3.5 m) on straw or wood shavings and had visual contact with conspecifics. Training and testing took place in an empty aisle adjacent to the aisles in which the stallions were kept. Horses had access to water *ad libitum*, and were not food restricted during the study; they were fed hay and concentrate three times a day. On a daily basis, horses were exercised (riding, carriage riding), allowed free movement on a sand paddock, or were walked in a horse walker.

### Rats

In total 16 male Lister-Hooded rats were obtained from Charles River Laboratories (Sulzfeld, Germany) after weaning at 21 days of age. Prior to the present study, the rats were used in another experiment investigating short-term positive emotional contagion (Lampe *et al*., manuscript in preparation) and were kept in groups of three in ‘Mickey 2 XL’ cages (l × b × h: 80 × 50 × 30 cm; Savic, Belgium). At 3 months of age they were re-housed in two groups of eight rats each. Rats were housed in large multi-level cages (l × b × h: 95 × 63 × 159 cm; ‘Suite Royale’, Savic, Belgium) with woodchip bedding (JRS Lignocel) and enrichment items such as tunnels, a running wheel, hammocks, and paper towels for nesting. They were housed under a 12:12 h reversed light:dark cycle (lights off at 09:00) and all training and testing procedures were carried out during the dark phase under red light. The test room was adjacent to the housing room and both rooms were maintained at 21–23 °C. Rats had access to tap water *ad libitum*, and were fed a restricted diet of standard rodent food (KLIBA NAFAG #3430, Switzerland) to maintain 90% of their free-feeding weight. Food was provided after training/testing. Rats were trained on the JBT in two batches at 8–9 months of age with each batch consisting of four rats from both cages.

### C57 and SWISS Mice

In total 24 female C57BL6/JRj (inbred strain, hereafter C57) and 24 female RjOrl:SWISS mice (outbred strain, hereafter SWISS) were obtained from Janvier Labs (Le Genest-Saint-Isle, France) after weaning at 21–25 days of age. All mice were part of a larger experiment investigating the effects of housing conditions on measures of animal welfare and variation in experimental results (Bailoo *et al*., manuscript in preparation). Due to the design of the above experiment, strains of mice were housed separately in groups of three under four different housing conditions, counterbalanced across batch and strain: (i) standard Type III cages (Tecniplast, Italy) with wood-chip bedding, (ii) bedding and nesting material, (iii) bedding, nesting, and structural enrichment, and (iv) large pet-sized cages (l × b × h: 80 × 50 × 30 cm; ‘Mickey 2 XL’, Savic, Belgium) with deep bedding, multiple nesting materials, a running wheel, and structural enrichment (Bailoo *et al*., in preparation). Mice were housed under a 12:12 h reverse light:dark cycle (lights off at 07:00) and all training and testing procedures were carried out during the dark phase under red light. The test room was adjacent to the housing rooms that were maintained at 21–23 °C. Mice had access to tap water *ad libitum*, and were fed a restricted diet of standard rodent food (KLIBA NAFAG #3430, Switzerland). Food restriction began 23 days before the start of this experiment. Mice were initially restricted to 85–90% of their free feeding body weight. As mice were still growing in the training period, their food allowance was increased over time by 1–3% over the five weeks of restriction with daily rations being provided after training/testing. Animals were trained on the JBT in two batches of 24 mice at 3.5 months of age; both batches were counterbalanced for strain.

### General principle of the novel Judgement Bias Task (JBT)

The JBT used here is a modification of common spatial Go/No-go task designs where the location of a single target (e.g. goal-box, bucket) is used to signal either reward (positive location) or non-reward (negative location). Animals are trained to approach the positive location (Go response) and to not approach the negative location target (No-go response; operationalised by a time-out value). Once the discrimination is learned, intermediate locations between the positive and negative locations are then presented as ambiguous cues, e.g.^25,26^. Unlike other spatial task designs, animals in our study were trained to initiate trials by performing an operant response, similar to Neave and colleagues^27^ who used a visual (rather than a spatial) task design. However, animals in their study were only trained to initiate trials after they had reached a certain criterion of correct positive (approach) and negative (no approach) trials, whereas in our study animals were trained to initiate trials before the discrimination training began. The advantage of our approach is that animals could learn to avoid waiting time in negative trials by having the option to immediately re-initiate a new trial.

In the present study, unlike previous spatial task designs, five goal-boxes were always present in the arena (one positive, one negative, and three ambiguous locations). These goal-boxes could be opened or closed manually by the experimenter (see Supplementary Information for more details). Consequently, it was not the location of the goal-box per se but the location of the open goal-box which served as the cue. Upon trial initiation, one goal-box was opened, and the animal could choose whether to approach the goal-box (Go response), to initiate a new trial (No-go response), or to wait until the maximum trial time was reached (again No-go response). Re-initiation and time-out trials were not differentiated for two reasons. Firstly, for horses and rats, time-outs rarely occurred after Stage 3 (see below), and secondly, due to the unprecedented speed of performance (see Results: Testing) of the mice (which performed both re-initiations and time-outs) the manual recording of only two response categories (Go or No-go responses) was possible. However, a more automated version of the same task design would allow for such differentiation also in mice. Since animals could initiate subsequent trials with virtually no inter-trial time, having all goal-boxes present minimised the need for any set-up of cues between trials. For example, closing and opening of *in situ* goal-boxes allowed for a more immediate presentation of the appropriate cue without having to move a single goal-box to a different location.

Once learning criterion was reached on the positive and negative goal-boxes (see below), animals were tested across six test sessions with ambiguous trials interspersed between the positive and negative trials. Go and No-go responses were recorded, and the ratio of Go to No-go responses to the ambiguous cues was used as measure of judgement bias.

### Overview of the apparatus/arena

**Figure 1:**
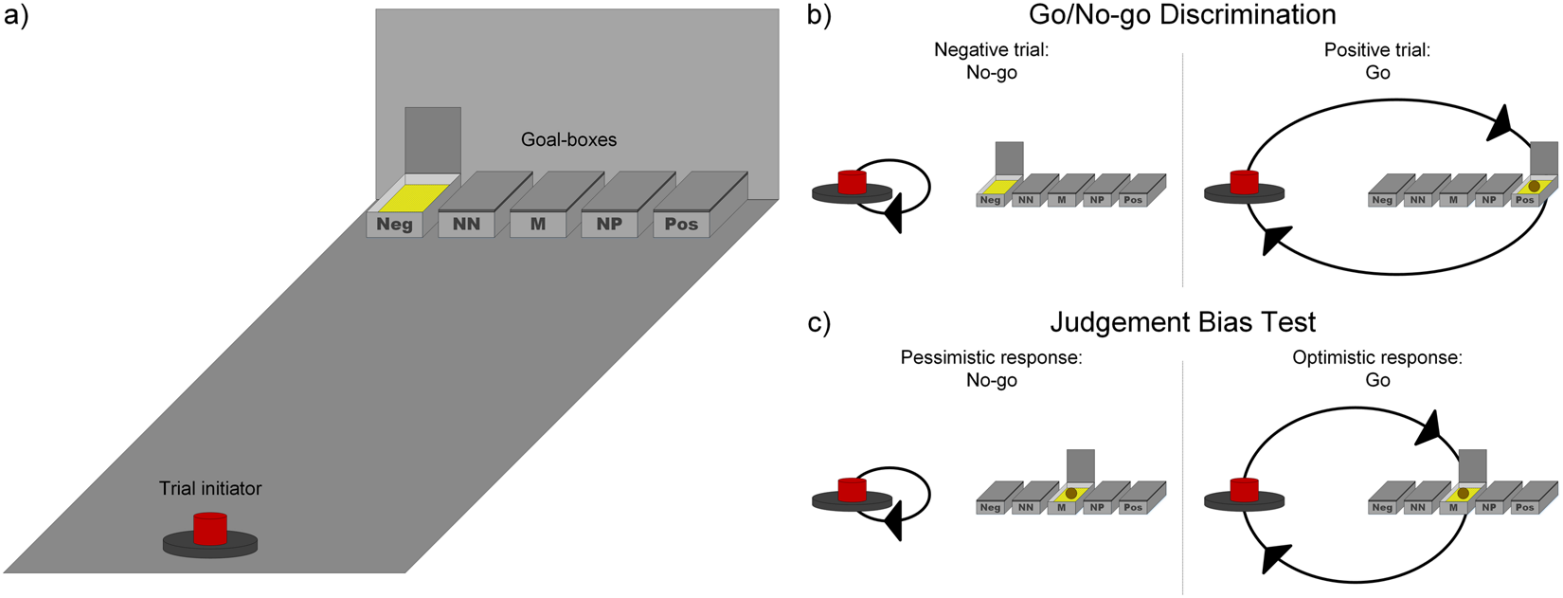
Schematic overview of the test apparatus and task design. Overview of the test arena with the trial initiator on one side and the five goal-boxes on the opposite side; Negative (Neg), Positive (Pos), and the three ambiguous locations, Near Negative (NN), Middle (M), and Near Positive (NP) (**a**). Correct responses as indicated by black arrows in the Go/No-go discrimination stage in positive (Go response) and negative (No-go response) trials (**b**). An ambiguous test trial and the two possible responses (Go and No-go response), indicated by the black arrows, as well as their interpretation (optimistic and pessimistic response, respectively) are illustrated (**c**).

Horses were trained and tested in an arena (Batch 1: l × b: 3.0 × 3.5 m; Batch 2: l × b: 5.3 × 2.95 m), rats and mice in a bespoke apparatus (rats: l × b × h: 60 × 50 × 40 cm; mice: l × b × h: 40 × 25 × 25 cm). The set-up of the different test systems is displayed and described in Supplementary Figs 1–3. However, the principle of the task design was the same for all species (Fig. 1).

The trial initiator was located on one side of the test arena, opposite to the five goal-boxes that were spaced equidistant from each other. Horses initiated trials by touching a plastic bottle suspended from the ceiling with their muzzle, whereas for the rodents, breaking the infra-red beam in a nose poke (H21-09R Nose Poke Operandum, Coulbourn Instruments, Holliston, MA, U.S.A.) served to initiate a trial. After a correct trial initiation (see below), the initiator was briefly made unavailable to indicate both correct trial initiation and the moment when a new trial could be initiated.

Upon trial initiation, a goal-box was opened manually by the experimenter behind the row of goal-boxes. The spatio-visual cue of the open goal-box was supported by additional auditory (a ‘clack’ of the opening lid of the goal-box) and visual (horses: salient colour pattern on the open lid, rodents: a light inside the open goal-box) cues.

#### Training and test protocol

Animals from each species were trained and tested in two non-overlapping batches, and the location of the positive and negative goal-boxes was counterbalanced across animals within each batch, but remained constant for each animal throughout training and testing. All animals were familiar with the food rewards before training on the JBT started. Training and testing followed a series of five stages (Table 2).

**Table 2:** Summary training and testing protocol.

Stage	Goal-boxes used	Rewarded goal-box	Trials per session	Criterion to proceed to next stage
Habituation	Pos	Pos	NA	Animal eating rewards when available
Shaping for Trial Initiation	Pos	Pos	NA	20 properly initiated trials followed by Go responses and consumption of reward
Left-Right Discrimination	Pos, Neg	Pos, Neg	50	80% correct Go responses to both sides within a single session
Go/No-go Discrimination	Pos, Neg	Pos	50	80% correct responses in positive (Go) and negative (No-go) trials across four consecutive 20-trial blocks
Judgement Bias Test	Pos, Neg, NN, M, NP	Pos, NN, M, NP	53	NA

### Training

#### Stage 1: Habituation

Animals were habituated to the arena/apparatus, to the opening and closing of the goal-box, and to finding food rewards in the positive goal-box. While all five goal-boxes were present throughout training and testing, only the designated positive goal-box was opened and baited at this stage. Habituation was deemed successful when animals were consistently eating the rewards when available.

#### Stage 2: Shaping for Trial Initiation

Following successful habituation, the animals were shaped to perform the operant response required for trial initiation (horses: touching the bottle, rodents: nose-poke) and a Go response in positive trials. Horses were first conditioned to touch the trial initiator with their muzzle. Delivery of the food rewards was then moved gradually further away from the trial initiator towards the positive goal-box. By contrast, rodents were gradually shaped to move increasingly away from the goal-boxes and towards the initiator to trigger the opening of the positive goal-box. Shaping was deemed successful when animals performed a session of at least 20 trials where the initiator was activated and was followed by Go responses and consumption of the food reward.

#### Stage 3: Left-Right Discrimination

Animals were trained to perform correct Go responses to both positive and negative goal-boxes across a series of sessions. This ensured that they were responding to the location of the open goal-box, and not simply focusing on whether the positive goal-box was open or not. Each session consisted of 50 self-initiated trials, pseudorandomly alternating between presentations of the positive and negative goal-box (maximum three consecutive trials on the same side, equal numbers of trials to the positive and negative goal-box per session), where both positive and negative goal-boxes were always rewarded. Approaches to the open goal-box within a specified length of time (horses: 10 s, rats and mice: 5 s) were considered a correct Go response and the animal obtained a food reward. Once the animal obtained the food reward, the goal-box was closed and the next trial began when the animal initiated a new trial. Contact with the closed goal-box before going to the open goal-box was considered an incorrect Go response for horses and rats (for mice see Supplementary Information). No-go responses were recorded if the animal did not approach the open goal-box within the specified time or if it initiated a new trial. Following an incorrect Go response or a No-go response, the goal-box was closed and the next trial began when the animal initiated a new trial. Once the animals performed 80% correct Go responses to both sides within a single session, they were ready to proceed to the next stage.

#### Stage 4: Go/No-go Discrimination

To teach the animals the contingencies associated with the positive and negative goal-boxes, they were trained across a series of sessions to perform Go responses in positive and No-go responses in negative trials. Each session consisted of 50 self-initiated trials, pseudorandomly alternating between positive (always rewarded) and negative (never rewarded) trials (maximum three consecutive positive or negative trials, equal numbers of positive and negative trials per session). Correct Go (hereafter Go responses) and No-go responses were defined as for the Left-Right Discrimination stage. Trials within sessions were split into three blocks, i.e. one 10-trial block and two 20-trial blocks, with each block containing equal numbers of positive and negative trials. Once the animals performed Go responses in 80% of positive trials and No-go responses in 80% of negative trials across four consecutive 20-trial blocks, they were ready to proceed to the test stage. The ten-trial blocks were considered as acclimatisation trials to remind the rodents of the task contingencies, and were therefore excluded from the criterion calculation. To keep the same learning criterion for the horses, and to ensure the same number of trials for data analysis, a 10-trial block was also excluded from the horse data.

### Testing

#### Stage 5: Judgement Bias Test

Judgement bias was assessed over six test sessions each consisting of 53 self-initiated trials; 25 positive and 25 negative trials, plus three ambiguous trials, whereby each of the three ambiguous cue locations (Near Positive (NP), Middle (M), and Near Negative (NN)) was presented once per session. Positive and negative reference trials were ordered according to the same pseudorandom rules as in previous stages, while ambiguous trials were always presented in trials 16, 32, and 48, but with the order being counterbalanced across the six sessions. Further, presentation of the same ambiguous cue occurred equally often (50%) after positive and negative trials. Since in our design, Go responses in positive trials always resulted in a reward, Go responses (as defined in Stage 3) to ambiguous goal-boxes were always rewarded (i.e. rewarded according to expectation) to reduce potential effects of surprising non-reward^28,29^. Go responses in ambiguous trials were interpreted as ‘optimistic’ responses, whereas No-go responses in ambiguous trials were interpreted as ‘pessimistic’ responses.

### Exclusion criteria

Criteria for the exclusion of animals in the course of the different stages of training were determined prior to the start of the experiments.

#### Habituation

Animals were excluded from further training if after two sessions, they showed severe signs of stress (horses: whinnying, running around, repeated defecation; rodents: escape attempts such as repeated jumping, or continued grooming/inactive behaviour) or, in case of the horses, if there was a risk of the animal hurting itself when trying to escape from the arena.

#### Shaping for Trial Initiation

Animals were excluded from the study if they did not perform (failure to eat rewards, failure to shape - move towards/away from trial initiator) during two consecutive sessions.

#### Left-Right Discrimination

Animals were excluded at this stage if they completed fewer than 50 trials in 45 minutes for three consecutive sessions.

#### Go/No-go Discrimination

Animals were excluded at this stage if they completed fewer than 50 trials in 45 minutes for five sessions. Moreover, due to time constraints, training was terminated if performance did not improve in the course of 20 sessions (horses) or 12 sessions (rodents), which by far exceeded the average number of sessions required for the rest of animals within a species or strain.

### Ethical considerations

This study was carried out in accordance with the guidelines of the Swiss Animal Welfare Ordinance (TSchV 455.1). The experiment with the horses was approved by the Cantonal Veterinary Office in Vaud, Switzerland (license number: 2804.1). The experiments with rats and mice were approved by the Cantonal Veterinary Office in Bern, Switzerland (license number rats: BE 17/13, license number mice: BE 16/16).

### Statistical analyses

#### Training

Training duration and attrition rate per species/strain and training stage are presented descriptively. Data are expressed in sessions (mean ± standard deviation (SD)) for the training duration and as number of animals for the attrition rate.

#### Testing

The results for the duration of the test sessions per species/strains are presented descriptively. All values are expressed in minutes (mean ± SD). To ensure stable performance of the animals in positive and negative trials during testing, we only included blocks of trials in the analyses in which animals displayed at least 7 correct responses out of 10 trials for both positive and negative trials. This criterion was defined based on the learning criterion (8 out of 10 positive and 8 out of 10 negative trials correct within one block, for learning it was for four consecutive blocks), allowing for one additional error each in positive and negative trials. Blocks that did not meet this criterion were dropped from further analysis since unstable performance towards the reference cues questions the validity of the responses towards the ambiguous cues^17^.

To investigate the effect of the five different trial types (Pos, NP, M, NN, Neg) on the animals’ decisions (binary outcome measure: Go response = 1, No-go response = 0), we used generalised mixed-effects models which adequately reflect dependencies within the experimental design (repeated measures, nested design). All models were run for each species/strain separately in R (version 3.3.2) using the function glmer of the package lme4 (‘family’: binomial, including the ‘logit’ link function^30^), and the alpha-level was set to 0.05.

In order to test whether animals showed a monotonic graded slope and thus differentiated between the five different trial types (Pos, NP, M, NN, Neg), models were run with ‘trial type’ as fixed effect. ‘Trial type’ was treated as continuous variable based on the suggestion of Gygax^17^. However, in order to specifically evaluate whether and how animals discriminated between the different trial types, models with ‘trial type’ as categorical variable were also run.

Random effects were ‘trial type’ nested in ‘session’ nested in ‘animal ID’ nested in ‘batch’. Since mice were tested twice daily, ‘session’ was replaced by ‘session per test day’ (1 or 2) and ‘test day’ (1–4) for both C57 and SWISS mice (‘trial type’ nested in ‘session per test day’ nested in’test day’, nested in ‘animal ID’ nested in ‘batch’). Table 3 gives an overview of the fixed and random effects and their levels in the case of categorical variables.

**Table 3:** Overview of all fixed and random effects, whether they were treated as continuous variable or as factor, and their levels (in case of factors).

Effect	Species/strains	Fixed or random	Type of variable	Levels
Trial type	all	fixed	continuous and categorical	when continuous: 1 (Pos), 0.75 (NP), 0.5 (M), 0.25 (NN), 0 (Neg) when categorical: Pos, NP, M, NN, Neg
Session x Trial type	all	fixed	both continuous	
Batch	all	random	categorical	1, 2
Animal ID	all	random	categorical	horses: 1–17* rats: 1–16 C57: 1–24 Swiss: 1–22*
Session	horses, rats	random	continuous	1–6
Test day	C57 & SWISS mice	random	continuous	1–4
Session per day	C57 & SWISS mice	random	categorical	1, 2
Trial type	all	random	continuous	

*Only animals that fulfilled the learning criterion and that were subsequently tested in the JBT are listed here.

Significant results of models with ‘trial type’ as factor were probed by pairwise *post hoc* analyses (function: glht, package: multcomp^31^). Comparisons of interest were always the two adjacent trial types, thus Pos-NP, NP-M, M-NN, NN-Neg, and p-values were adjusted for multiple testing by a single-step method incorporating the correlations between the test statistics^32^.

To assess the effect of session on decisions in ambiguous trials, we analysed whether the number of Go responses in these trials increased over the course of the test sessions. To this end, the interaction between ‘session’ and ‘trial type’ was analysed. Again, models were run for all species/strains, and random effects remained the same as described above.

### Data availability

Raw data for all outcome measures will be made available upon request.

## Results

### Training

#### Training duration

Horses needed between 5 and 19 (10.6 ± 4.3), rats between 3 and 7.5 (4.3 ± 1.2), C57 mice between 3 and 7 (4.5 ± 1.1), and SWISS mice between 3.5 and 12 (6.6 ± 2.0) sessions in the Go/No-Go Discrimination stage to reach criterion for testing. The number of sessions needed for Habituation, Shaping for Trial Initiation and Left-Right Discrimination is presented separately in Table 4 since the sessions are not directly comparable between species (see Supplementary Information).

**Table 4:** Training duration (number of sessions ± SD) of all species/strains with regard to the different training stages.

Training Stage	Horses		Rats		C57 Mice		SWISS Mice	
	Batch 1	Batch 2	Batch 1	Batch 2	Batch 1	Batch 2	Batch 1	Batch 2
Habituation	^2^	^2^	6.3 ± 0.5	4.0 ± 0	1.9 ± 0.3^1^	1.0 ± 0^1^	1.6 ± 0.5^1^	1.0 ± 0^1^
Shaping for Trial Initiation	4.7 ± 1.2^2^	2.1 ± 0.7^2^	6.4 ± 0.9	6.0 ± 3.4	6.9 ± 1.4	7.7 ± 1.2	6.1 ± 1.5	7.2 ± 1.3
Left-Right Discrimination	2.9 ± 1.1^3^	1.1 ± 0.5^3^	1.0 ± 0	1.5 ± 1.4	1.0 ± 0	1.0 ± 0	1.0 ± 0	1.0 ± 0
Go/No-go Discrimination	8.8 ± 3.2	12.7 ± 4.7	4.5 ± 1.3	4.1 ± 1.1	4.3 ± 1.2	4.7 ± 1.1	6.3 ± 2.0	6.9 ± 2.0

^1^As mice were already familiar with the apparatus and only the back wall was novel, only minimal habituation was needed.

^2^Habituation and Shaping for Trial Initiation combined.

^3^Differently sized arenas for horses of Batch 1 and Batch 2 (see Supplementary Information).

#### Attrition rate

Individual animals were excluded at different training stages if they did not fulfil the criteria specified above. An overview of the number of discarded individuals at each stage is given in Table 5.

**Table 5:** Number of discarded individuals per training stage, species/strain, and batch.

Training Stage	Horses		Rats		C57 Mice		SWISS Mice	
	Batch 1	Batch 2	Batch 1	Batch 2	Batch 1	Batch 2	Batch 1	Batch 2
Habituation	1	0	0	0	0	0	0	0
Shaping for Trial Initiation	1	4	0	0	0	0	0	0
Left-Right Discrimination	0	0	0	0	0	0	0	0
Go/No-go Discrimination	1	1	0	0	0	0	1	1

### Testing

#### Descriptives of performance at testing

Most of the animals learned the discrimination and were subsequently tested (horses: 17/25, rats: 16/16, C57: 24/24, SWISS: 22/24). On average, a test session of 53 trials (25 positive, 25 negative and 3 ambiguous trials), lasted for 14.1 ± 4.9 minutes, with horses taking 18.2 ± 5.3 minutes, rats taking 8.7 ± 2.3 minutes, C57 mice taking 17.1 ± 5.7 minutes, and SWISS mice taking 13.5 ± 6.0 minutes. Table 6 gives an overview of the number of blocks that were excluded per species/strain due to making more than three errors per block in positive and/or negative trials.

**Table 6:** Overview of the number of blocks that were excluded per species/strain, and batch.

	Block	Horses		Rats		C57 Mice		SWISS Mice	
		Batch 1	Batch 2	Batch 1	Batch 2	Batch 1	Batch 2	Batch 1	Batch 2
Positive trials	1	1	1	0	0	0	0	0	0
	2	1	4	0	0	0	0	0	0
Negative trials	1	0	8	2	2	3	0	3	3
	2	8	13	1	4	6	0	4	4
Excluded blocks (sum)	1 & 2	10	25^1^	3	6	9	0	7	7
Excluded blocks (%)	1 & 2	9.3	26.0	3.1	6.3	6.3	0	5.3	5.3

^1^One block was represented twice because the criterion of making more than three errors was reached for both positive and negative trials.

#### Discrimination of the different trial types

**Figure 2:**
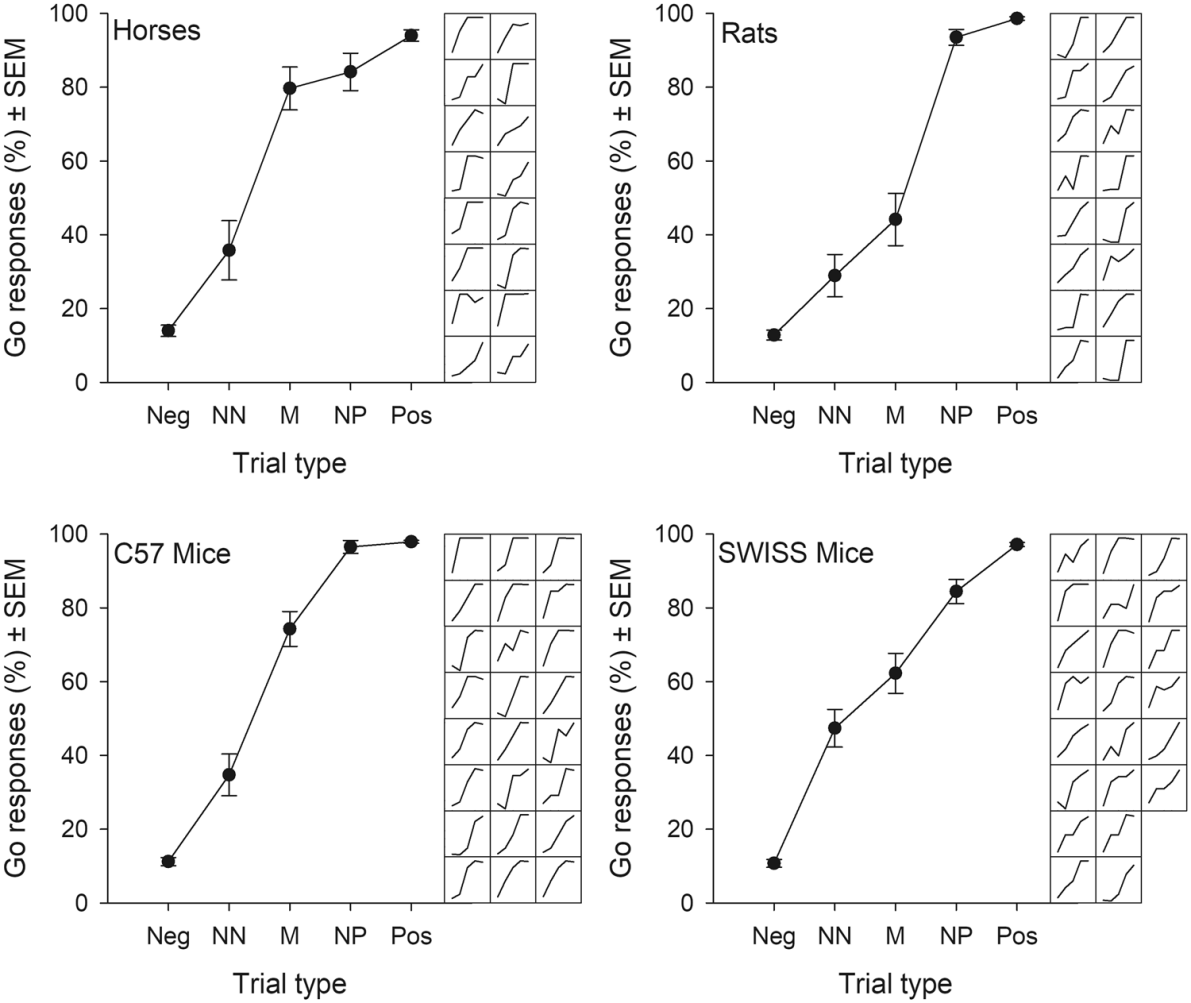
Percentage of Go responses per group and individual across trial type at testing. Mean percentage of Go responses ± standard errors of the mean (SEM) are shown for each species/strain for all trial types (positive, negative and ambiguous trials). The data are also presented at the individual level per species/strain alongside each group level graph.

Animals of all species/strains differentiated between the different trial types as assessed by graphical evaluation of the slopes both at the group (species/strain) and at the individual level (Fig. 2), as well as via statistical confirmation when ‘trial type’ was treated as either a continuous or categorical variable (Table 7). Results of *post hoc* tests indicating how animals differentiated between the pairs of adjacent trial types are presented in Table 8.

**Table 7:** Results of the generalised linear mixed-effects models with ‘trial type’ as continuous and categorical variable.

Species/Strain	Trial type	Test statistic	P-value
Horses	continuous	χ^2^_1_ = 465.90	<0.001
	categorical	χ^2^_4_ = 477.44	<0.001
Rats	continuous	χ^2^_1_ = 545.82	<0.001
	categorical	χ^2^_4_ = 587.82	<0.001
C57 Mice	continuous	χ^2^_1_ = 839.02	<0.001
	categorical	χ^2^_4_ = 844.91	<0.001
SWISS Mice	continuous	χ^2^_1_ = 649.56	<0.001
	categorical	χ^2^_4_ = 663.33	<0.001

**Table 8:** Pairwise comparisons of the animals’ responses to adjacent pairs of the five trial types (Pos, NP, M, NN, Neg) with the significant results highlighted in bold.

Adjacent trial types	Horses		Rats		C57 Mice		SWISS Mice	
	z-score	p-value	z-score	p-value	z-score	p-value	z-score	p-value
Pos-NP	3.5	**0.004**	3.48	**0.004**	1.29	0.672	6.48	**<0.001**
NP-M	0.84	0.911	6.31	**<0.001**	4.56	**<0.001**	4.08	**<0.001**
M-NN	−5.88	**<0.001**	−2.23	0.153	−6.71	**<0.001**	−2.47	0.091
NN-Neg	4.28	**<0.001**	4.13	**<0.001**	7.65	**<0.001**	10.69	**<0.001**

#### Effect of session on decisions in ambiguous trials

**Figure 3:**
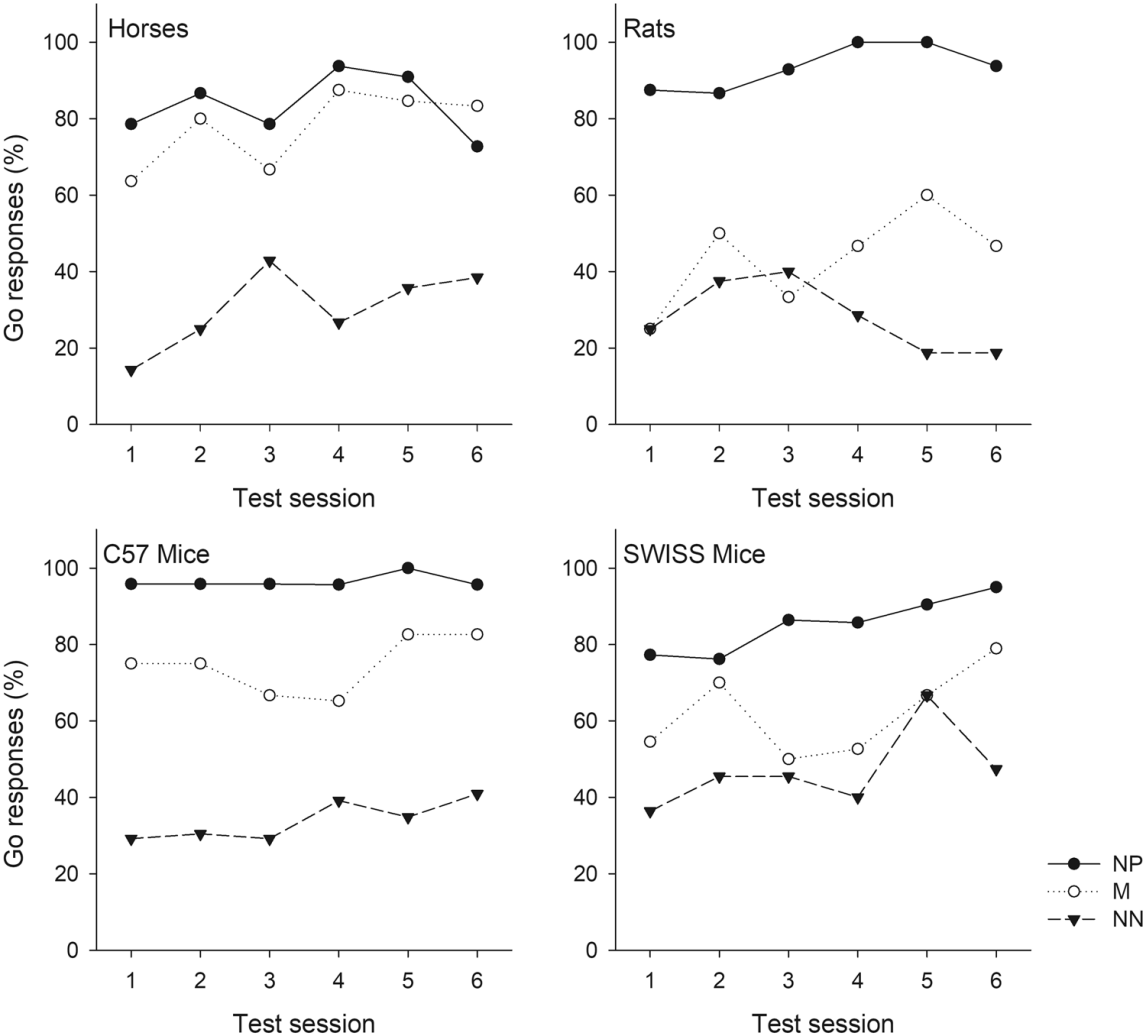
Percentage Go responses per ambiguous trial type across the six test sessions. The percentage of Go responses per ambiguous trial type (NP, M, NN) is presented for each species/strain across all six test sessions. Please note, the three ambiguous trial types are dependent within each test session, but for visual clarity are presented here as separate lines.

The number of test sessions (1–6) did not have an effect on the animals’ responses in the ambiguous trials as indicated by the results for the interactions between ‘session’ and ‘trial type’ (horses: χ^2^_1_ = 0.36, p = 0.550, rats: χ^2^_1_ = 0.00, p = 0.951, C57: χ^2^_1_ = 2.28, p = 0.131, SWISS: χ^2^_1_ = 0.79, p = 0.376, Fig. 3).

## Discussion

This study aimed to test a Judgement Bias Task (JBT) design for non-human animals in terms of whether it can be applied successfully across different species/strains of mammals, is relatively quick to learn, produces monotonic graded responses to the different ambiguous cues, and no learning about the outcome of ambiguous cues occurs during testing. We demonstrated that the same task design, with only few species-specific modifications, and the same protocol, could be applied successfully to horses, rats, and two strains of mice. All species learned to Go for positive cues and No-go for negative cues (unlike^12^). Training was relatively quick for all species/strains and, on the group level, all species showed monotonic graded responses from the negative to the positive cues, indicating that the animals discriminated between the different trial types. While not all individuals showed a monotonic graded response across the different trial types, in general inter-individual variation was relatively low considering the small number of ambiguous cues presented at the individual level (max. six per trial type). Even though Go responses to ambiguous trials were always rewarded, and were presented six times each, no evidence of learning about the outcomes of ambiguous trials was found across the six test sessions. Therefore, while this task design requires further validation with respect to the detection of judgement biases, it represents a promising first step towards the implementation of a practical judgement bias task that works across species.

In the current study, we aimed to take advantage of the fact that some task designs are easier to learn than others (Go/No-go easier than Active Choice tasks, e.g.^8,15^; spatial stimuli easier than other cue modalities, e.g. visual cues^33^). In our task, the spatial cues were further enhanced with visual and auditory signals to promote learning. The spatial cue and additional signals were features of the goal-boxes themselves and thus should have some degree of ‘unconditioned significance’^34^, i.e. the cue is significant without requiring ‘artificial’ associations to be learned - as opposed to designs where there is no intuitive relationship between the cue and the outcome, e.g. use of tone cues^15,22^. We also provided an element of control in the form of active trial initiation. Thus, this design aimed to incorporate certain easy-to-learn features typical of spatial Go/No-go tasks, with the advantage of always requiring an active response by the animal through trial initiation.

Most of the animals from the different species/strains investigated in this study performed a large number of trials per session in a short amount of time, thus reducing the number of sessions required to learn the discrimination (see Introduction, Hypotheses 1 & 2). Comparing training duration across studies is difficult since learning criteria have varied considerably, e.g.^11^. Training duration also depends on task design (e.g. Go/No-go versus Active Choice tasks), cue types (e.g. spatial, visual or auditory), and outcome measures (e.g. latency versus choice-based tasks) additionally making comparisons difficult. If we are interested in a feasible task design, we could consider experimenter time (roughly estimated by the number of sessions to reach criterion on the Go/No-go discrimination) as the metric to evaluate this. With this in mind, we find that, within species, the number of sessions to learn the Go/No-go discrimination is comparable to previous spatial Go/No-go designs (ranges of sessions to criterion in previous studies; horses: 3–11^35–37^ rats: 2–7^18,21,38^; mice: 5–9^26^) despite the more complex nature of the response required in the present task (active No-go response). Compared with Active Choice tasks, training duration was considerably shorter in our task (sessions to criterion in Active Choice tasks; horses: 41 sessions but only one horse reached the testing stage^15^; rats: sessions to criterion rarely reported, but 8–26^39^ mice: 14 (Swiss mice^16^) and 20 (C57 mice^16^)).

While we have to acknowledge that the Shaping for Trial Initiation stage in the present design increases experimenter time, we believe that the benefits of active trial initiation, discussed below, outweigh the extra time needed for shaping. On top of this, our task has the advantage of a very high reference:ambiguous trial ratio (50:3, see below for comparison with other studies), and in general a strict criterion required to reach the testing stage. Further, in traditional Go/No-go tasks correct responses in negative trials (i.e. No-go responses) cannot be differentiated from omissions. Whereas we did not differentiate between No-go responses and omissions in the present study, the design does allow for this evaluation in future studies.

An average test session of 53 trials lasted less than 15 minutes across species/strains, reflecting a relatively high number of trials in a relatively short time (approximately 3.5 trials per minute). As with training duration, it is difficult to compare session duration across studies since this information is not reported in most publications. However, a comparison of the number of trials per session across studies revealed that 53 trials is a rather high number of trial presentations. All previous studies on mice^12,16,26,40^ and horses^15,35–37^ included considerably fewer trials per session (mice: range from 1–32 trials; horses: range from 7–13 trials), and only some studies on rats exceeded 53 trials, e.g. with a maximum of 100 trials per session^41^. Considering the ratio of trial number to session duration, we can compare our results with the data from our own previous studies, in which one test session of 13 trials took on average 25 minutes for rats (unpublished data) and 30 minutes for horses^15^, both tasks using auditory cues. In the current study, the higher number of trials per session obtained is primarily due to the short duration of negative trials, since animals in these trials could just re-initiate a new trial instead of waiting for a predetermined time before a new trial started. In addition, the design of the task precluded any need for set-up between trials, further reducing the session length by eliminating inter-trial time intervals, and for the horses, the test arena was physically smaller than those used in previous studies^15,35–37^, reducing the time required to make a Go response. Training duration was thus reduced considerably for both session length and the number of sessions to reach criterion (the latter at least partly due to the high number of trial presentations within one session).

An important aim of this study was to assess the validity of the task design with respect to the theoretical assumptions of such tasks. To this end, we examined whether animals’ responses across the different trial types represented a monotonic graded pattern from negative to positive trial types^17^. Such a pattern demonstrates that animals interpret the ambiguous cues with reference to the positive and negative cues and not simply as novel stimuli. Flat response curves have been found in previous studies, e.g.^15,18,19^ and question the validity of these task designs. In our study, animals of all species/strains showed graded responses to the different trial types as shown both graphically and statistically (see Introduction, Hypothesis 3). Specifically looking at the differences between each pair of adjacent trial types (Pos-NP, NP-M, M-NN, NN-Neg), we showed that animals generally discriminated between all but one pair of adjacent trial types, and this pair differed between species/strains. Although not always significantly different, mean Go responses to adjacent trial types always increased across the cues from negative to positive as expected. Moreover, we could show that this monotonic graded response pattern was present at the individual level for many of the animals resulting in the relatively low variation with respect to each ambiguous trial type (Fig. 2). Such low variation within trial type could render this design more sensitive to treatment differences which influence emotional state and thus judgement of the ambiguous stimuli.

Despite this promising response pattern for all species/strains, the stability of performance with respect to positive and negative trials during testing was not equal across species. Whereas the number of excluded blocks was extremely low for the rodents, it was higher for the horses, likely because of their low motivation (as discussed below). However, since a stable performance towards the positive and the negative cues is crucial for the interpretation of responses towards the ambiguous cues^17^, in an ideal task, ambiguous trials would only be presented when an animal is demonstrating consistent performance in the reference trials. Such a flexible system would preclude any need to exclude ambiguous responses from the analysis. When this is not possible, we would encourage scientists to specifically report the error rate in their studies.

One major limitation of the JBT paradigm is that there is little knowledge on what animals may learn about ambiguous cues with repeated presentations. If animals learn about the outome (i.e. reward or no reward) associated with the ambiguous cues, this would compromise ambiguity perception^9^. In repeated testing with non-rewarded outcomes, Doyle and colleagues showed that sheep approached non-rewarded ambiguous locations significantly less often over the course of nine test sessions^42^, while pigs in an Active Choice design took longer to respond and made more omissions in response to repeated presentations of non-rewarded ambiguous cues^22^. This evidence suggests that animals quickly learned that the ambiguous locations were not rewarded. In contrast to this and many other studies, we rewarded Go responses in ambiguous trials since we had experienced previously that animals showed signs of frustration when they performed a Go response in ambiguous trials, reflecting their expectancy of a reward, but did not receive anything, e.g.^15^. There was no indication in the present study that animals learned that Go responses in ambiguous trials were always rewarded since ‘optmistic’ responses did not increase across the six test sessions (see Introduction, Hypothesis 4). One explanation for this result might be the relatively high ratio of reference trials to ambiguous trials (50:3) within a test session. A similar result was shown in a study by Deakin and colleagues^43^ on laying hens with a ratio of reference trials to ambiguous trials of 40:6 within a session. As we had rewarded go responses in ambiguous trials while Deakin *et al*. had not, and both of these studies had used a relatively high number of test sessions (n = 6) compared to other studies (based on review by Gygax^17^: horses and mice: 1–4 sessions, rats 1–10 sessions), we conclude that the high ratio of reference cues to ambiguous cues may be the best way to minimise the risk of learning about the outcome of ambiguous trials. With this design, more data about responses to ambiguity can be collected, rendering our test less sensitive to day-to-day variation. It remains to be studied at what point animals learn about the outcomes of ambiguous trials in such a design.

With respect to the applicability of our design to different species, some issues should be taken into account. Whereas all rats and C57 mice successfully learned the task, and only two SWISS mice out of 24 individuals were discarded – a lower attrition rate than in other studies where this information has been reported (rats: 5/16^44^; mice: 3/12–7/12^26^, 2/16 and 4/16^16^) - the proportion of discarded horses (8/25) was much higher. However, of the animals that reached the Go/No-go Discrimination, only two failed to learn, resulting in an attrition rate of 2/19 horses at this stage. Consequently, if reasons for non-performance at the previous stages could be addressed, we would expect a low attrition rate similar to that of the rodents. To our knowledge, all JBT studies with animals have used food as the positive reinforcer. It is therefore essential that the animals are motivated to obtain the reward. Motivation can be manipulated by varying the quality of the reward (e.g. M&Ms® for pigs^22^) and by the use of food restriction, e.g.^16^. In our study, rodents underwent a mild (rats) to moderate (mice) food restriction and were rewarded with a highly palatable chocolate-flavoured pellet for correct responses in positive trials. Instead, the feeding regime of the horses could not be manipulated in this study due to facility restrictions, and piloting (data not shown) with different combinations/types of reward showed that the standard food was preferred by the horses and was thus used as the reward. Therefore, it is most likely that the lack of food restriction explains the horses’ reduced performance compared to all other animals. This was also reflected by horses having (i) a higher attrition rate in the Shaping for Trial Initiation stage, (ii) a larger number of sessions needed to acquire Go/No-go Discrimination, (iii) a poorer maintenance of the learning criterion during test sessions, and (iv) errors in positive trials and more errors in the 2^nd^ half of the session, compared to the rodents. Since horses made more errors in the second half of sessions, it could be that without food restriction, requiring animals to perform a high number of consecutive trials per se might also affect motivation^45^. Since this task design requires active trial initiation, it relies strongly on the animals being motivated to participate. Food restriction is commonly used to manipulate motivation in laboratory rodents, and it is possible that the restriction itself could affect the animals’ responses in the JBT as it has been suggested in sheep, whereby chronically restricted sheep tended to show a more optimistic bias in a Go/No-go task than sheep on a high feeding level^46^. However, laboratory mice and rats are considered to be metabolically morbid^47^; restrictions of 20–40% below ad libitum feeding improved long-term health and longevity in mice^47^. It remains to be investigated whether improved physical health deriving from a restricted diet can relate to (positive) judgement bias. In our study, factors other than food motivation (e.g. age: Batch 1 horses were younger than Batch 2 horses; larger arena size in Batch 2) might also have interacted with motivation as the performance of Batch 1 and Batch 2 horses was not equal.

The implementation of the active trial initiation in the early stages of training allowed the animals to have control over the presentation of trials, and although this leaves the task dependent on the animals’ motivation, it is likely to have prevented frustration during No-go trials. Instead of having to wait for a fixed time interval before the next trial could start, as it has been the case in the majority of previous Go/No-go studies, e.g.^13,14^, animals could immediately initiate the next trial when confronted with the negative cue. It has been shown that animals have a preference for control over no control^48^, and that they find it rewarding to exercise control over their environment^49^. Control is discussed as a ‘major adaptive aspect of the animal’s behaviour’^50^. Lack of control has been suggested to represent a key factor in the causation of a stress response^51^ and is the main concept of the learned helplessness paradigm used to model depression^52^. Instead, by being given the opportunity to actively participate in their environment, animals are able to effect change as they are given control over the situation^50^. Both human and non-human individuals with (perceived) control over a situation perform generally better than individuals in uncontrollable situations (reviewed in^53^). The introduced element of control in our study may thus have substantially contributed to the good learning performance of the animals in this study.

Furthermore, active trial initiation might have reduced the number of incorrect responses due to inattention or distraction by increasing the focus of the animals on the task contingencies, in the same way as perceived control over an outcome is associated with engagement and achievement in children^54^. This aspect is especially advantageous when animals are trained in a busy and noisy environment, which is often the case with horses and other farm animals. Additionally, by initiating their own trials animals returned to the start position by themselves after each trial. This was not only a practical benefit in terms of saving time, but it also reduced the direct interaction between animal and experimenter and thus potential unintentional biases caused by the experimenter on the task outcome.

In conclusion, this was the first study in which animals from three different species and different strains were successfully trained and tested using the same JBT design, a spatial task design with active trial initiation, and training protocol. Whereas the JBT paradigm needs more validation in terms of construct/predictive validity, having a task design that is relatively fast to train across species/strains, meets the theoretical assumptions in terms of monotonic graded responses, and allows for a high number of presentations of ambiguous cues, is a promising step towards this validation process.

## Electronic supplementary material

Supplementary Information
