# Supplementary material for: A cross-species judgement bias task: integrating active trial initiation into a spatial Go/No-go task
Source: Sci Rep. 2018 Mar 23;8:5104. doi: 10.1038/s41598-018-23459-3 (PMC5865189; doi:10.1038/s41598-018-23459-3)
Supplement: Supplementary file 1 — Supplementary Information [file 41598_2018_23459_MOESM1_ESM.pdf]

A CROSS-SPECIES JUDGEMENT BIAS TASK: INTEGRATING ACTIVE TRIAL INITIATION INTO A SPATIAL GO/NO-GO TASK

Sara Hintze, Luca Melotti, Simona Colosio, Jeremy D. Bailoo, Maria Boada-Saña, Hanno Würbel, Eimear Murphy

SUPPLEMENTARY INFORMATION: SPECIES-SPECIFIC INFORMATION

APPARATUS DESIGN

Summary information per species is provided in Supplementary Table S1 and more detailed descriptions in the following sections. As the apparatus used was the same for both C57 and SWISS mice, the information on these animals is not divided by strain and instead presented for the mice in general.

Horses

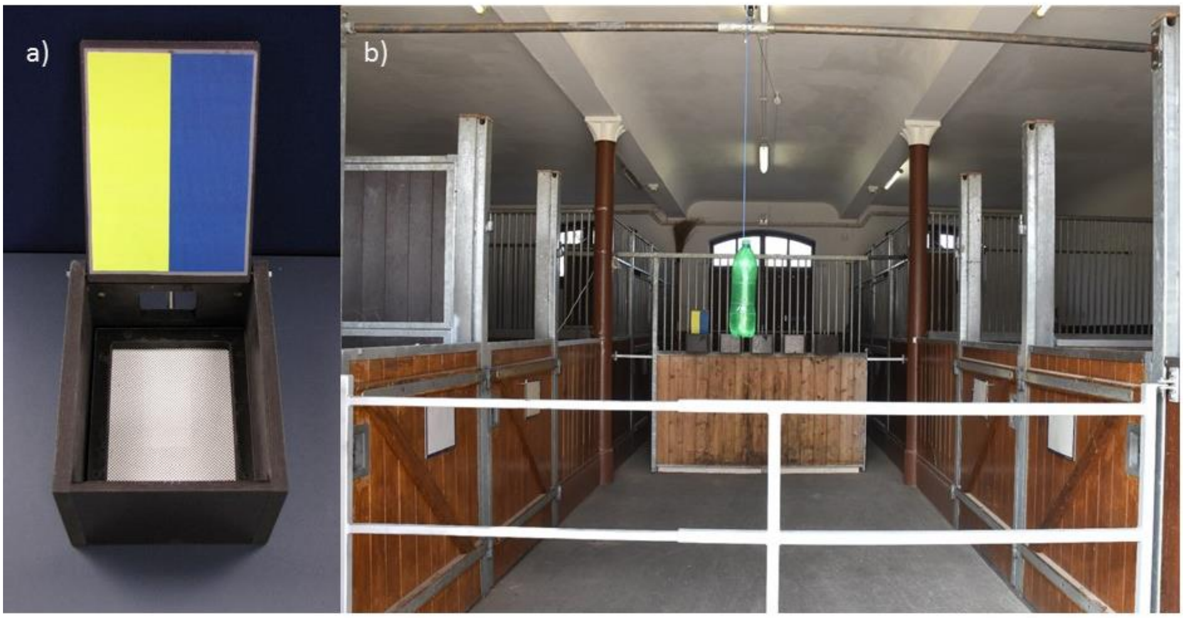

Supplementary Fig S1. Goal-box and test arena of the horses.
An open goal-box (a) that served as the cue for training and testing horses and the test arena viewed from behind (b) with the five goal-boxes mounted at the front wall of the arena and the trial initiator (green plastic bottle) suspended from the ceiling at the back of the arena.

Horses were tested and trained under natural light in a test arena situated near to the home stables. The arena differed in size between Batch 1 (l x b: 3.0 x 3.5 m) and Batch 2 (l x b: 5.3 x 2.95 m). The size was increased because space limitations may have interfered with performance of horses in Batch 1. The trial initiator was suspended from the ceiling at the back of the arena, while five identical rectangular wooden goal-boxes (l x b x h: 27 x 18 x 14 cm) were mounted 10.75 cm (Batch 1) or 14 cm (Batch 2) apart and at a height of 1.31 m (Batch 1) or 1.25 m (Batch 2) at the front of the arena

(Supplementary Fig S1 for Batch 2 set-up). The experimenter operated the opening of the goal-boxes and the movement of the trial initiator from behind the wall with the goal-boxes.

The goal-boxes served as spatial cues, but the blue and yellow pattern on the inner side of the lid (same pattern in each goal-box) that was only visible when the goal-box was open, and the 'clack' sound when opening the lid served as additional visual and auditory cues, respectively. Each goal-box could only be opened and closed manually by the experimenter rotating a metal stick connected to the lid. The open lid signalled the presence or absence of reward contained within the goal-box. The outermost goal-boxes were designated as the positive (always rewarded) and negative goal-boxes (never rewarded), while the three intermediate goal-boxes were designated as the ambiguous goal-boxes (Near Positive (NP), Middle (M), Near Negative (NN)). Within each goal-box, a perforated floor concealed inaccessible rewards to prevent odour cues from indicating the presence or absence of reward. A hole at the back of the goal-box allowed for reward delivery (one level tablespoon of concentrate (Hypona 788, UFA AG, Switzerland) mixed with oats (full grain, Landi, Switzerland) which was the usual food).

The trial initiator was a green plastic 2 l bottle filled with approximately 0.5 l of water suspended from the ceiling at a height of 1.45 m, which could be raised and lowered by the experimenter. If the animal touched the bottle with its nose/head, the bottle was raised out of reach for two seconds. This raising of the bottle indicated a correct trial initiation and allowed the horse to focus on the goal-box cues before making a decision either to go or to re-initiate.

Rats

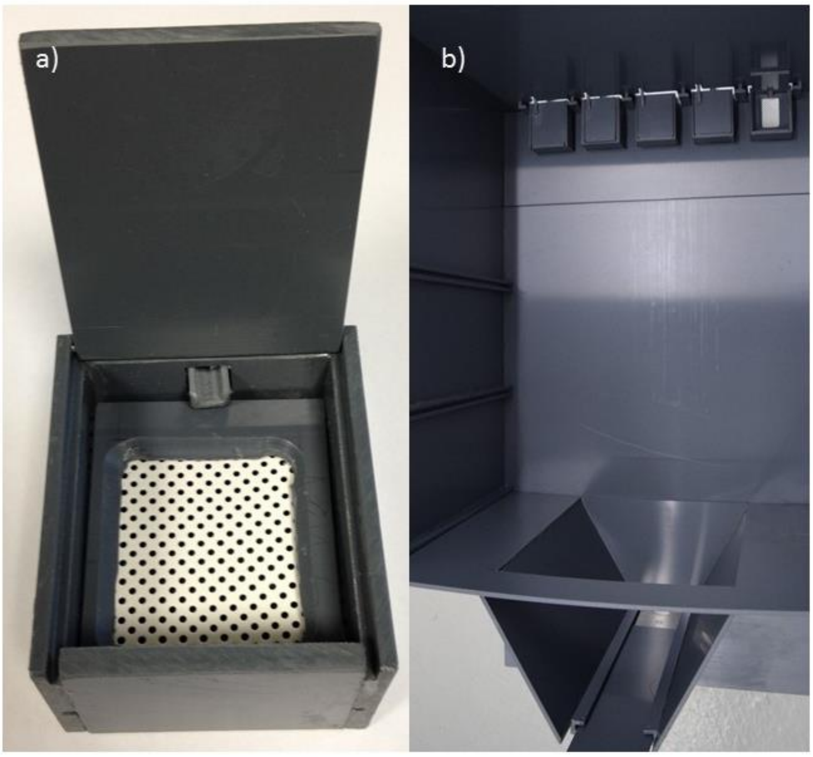

Supplementary Fig S2. Goal-box and test apparatus of the rats.
An open goal-box (a) that served as the spatial cue when training and testing rats and an overview of the test arena (b) with the five goal-boxes at the top and the nosepoke used as trial initiator at the bottom.

Rats were tested and trained under red light in an apparatus situated in a test room adjacent to the housing room. The apparatus consisted of a rectangular test arena (l x b x h: 60 x 50 x 40 cm) with an additional V-shaped area at one end (depth 15 cm) containing the trial initiator and a row of five identical rectangular goal-boxes (l x b x h: 6 x 5 x 4 cm) 2 cm apart from each other at the other end (Supplementary Fig S2), resulting in a distance of 69 cm between the initiator and the central goal-box. The experimenter operated the apparatus manually from behind the goal-boxes by pressing a short lever behind the respective goal-box to open it. Water was available *ad libitum* via two water bottles attached at the sides of the arena.

The goal-boxes served as spatial cue, but a white LED (connected to a 9v power source) placed underneath the perforated floor of the goal-boxes and the ‘clack’ sound when opening the lid served as additional visual and auditory cues, respectively. Each goal-box could only be opened by the experimenter pressing a lever from behind. The open lid signalled the presence or absence of reward contained within the goal-box. The outermost goal-boxes were designated as the positive (always rewarded) and negative goal-boxes (never rewarded), while the three intermediate goal-boxes were designated as the ambiguous goal-boxes (Near Positive (NP), Middle (M), Near Negative (NN)). Within

each goal-box, a perforated floor concealed inaccessible rewards to prevent odour cues from indicating the presence or absence of reward. A small chute at the back allowed for delivery of reward into the goal-box (45 mg chocolate flavoured Dustless Precision Pellets®, Bio-Serv, New Jersey, U.S.A.).

The trial initiator was a nosepoke operandum (H21-09R, Coulbourn Instruments, Massachusetts, U.S.A.), connected to the Habitest Modular System® (Coulbourn Instruments), with an opening of 2.5 cm in diameter. The nosepoke contained an infrared (IR) beam and pairs of red, green, and yellow LEDs, of which only the green and yellow were used. The nosepoke was programmed so that by default both the yellow and green sets of LEDs were on, signalling that the initiator was available. If the animal broke the IR beam with its nose, all LEDs switched off to signal a correct initiation and that the initiator was briefly deactivated. One second after the animal had removed its nose from the nosepoke, the green LEDs switched on to signal that the initiator was available again to initiate a new trial, while the yellow LEDs instead flashed at 4 Hertz for a further 4 s before remaining on to signal to both the animal and the experimenter the maximum trial length (5 s). If an animal remained with its nose in the nosepoke, or repeatedly nose-poked before the 1 s time-out was up, the initiator stayed inactive until the animal had removed its nose for 1s. The 1 s time-out should allow the animals to focus on the goal-box cues before making a decision either to go or to re-initiate.

Mice

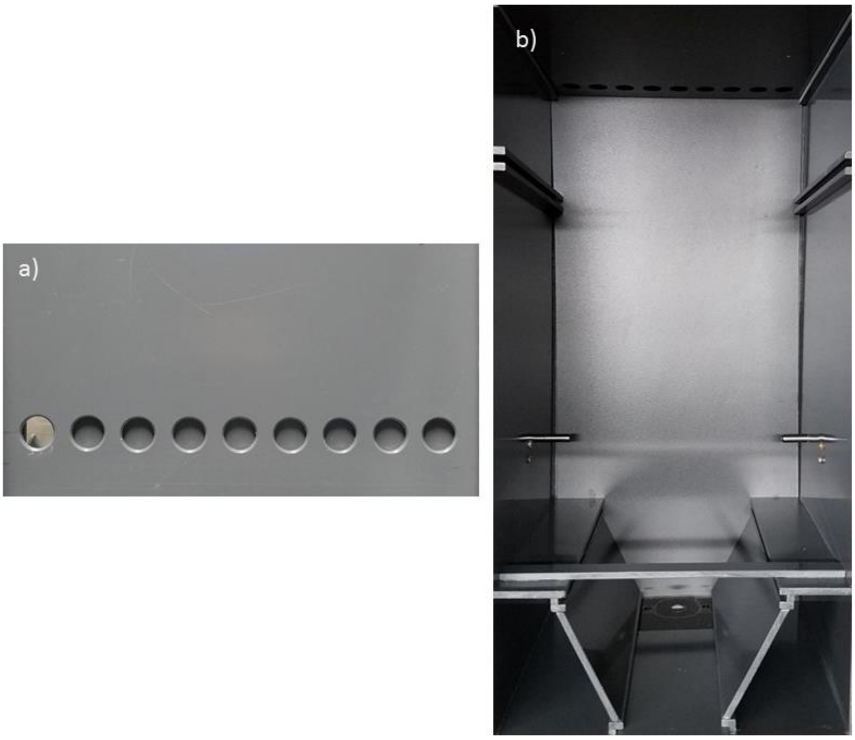

Supplementary Fig S3. Goal-holes and test apparatus of the mice.
The row of holes or goal-boxes (a) that served as the spatial cue when training and testing mice. Only five of the holes were used in the current study: every second hole starting from the outmost hole. Overview of the test arena (b) with the goal-holes at the top and the nose poke apparatus used as trial initiator at the bottom.

Mice were tested and trained under red light in three virtually identical apparatuses situated in a test room adjacent to the housing rooms. Each apparatus consisted of a rectangular test arena (l x b x h: 40 x 25 x 25 cm) with an additional V-shaped area at one end (depth 10 cm) containing the trial initiator and a row of nine identical goal-holes (Supplementary Fig S3), resulting in a distance of 50 cm between the initiator and the central goal-box. Of these nine goal-holes (⌀ 1.5 cm, 3.7 cm apart from each other and beginning 2.5 cm above the apparatus floor) only five were used for the purpose of the current experiment. Each goal-hole was blocked by a sliding door which the experimenter could slide upwards to reveal the hole. A barrier above each hole ensured that doors could be opened quickly but would remain in their guides. The experimenter operated the apparatus from behind the goal-boxes. Water was available *ad libitum* via two water bottles attached at the sides of the arena.

The goal-holes (hereafter goal-boxes) served as the spatial cue in the task, but a white LED (connected to a 9v power source) placed behind each hole and the ‘clack’ sound when the door hit the barrier served as additional visual and auditory cues, respectively. Each goal-box could only be opened by the experimenter by sliding the door upwards. The open door signalled the presence or absence of reward

behind the goal-box. The outermost goal-boxes were designated as the positive (always rewarded) and negative goal-boxes (never rewarded), while the three intermediate goal-boxes were designated as the ambiguous goal-boxes (Near Positive (NP), Middle (M), Near Negative (NN)). A small chute at the back allowed for delivery of reward into the goal-box (20 mg chocolate flavoured Dustless Precision Pellets®, Bio-Serv, New Jersey, U.S.A.). No odour control was necessary as rewards were only delivered after the animal had made its choice.

The trial initiator was a nosepoke operandum (H21-09R, Coulbourn Instruments, Massachusetts, U.S.A.), connected to the Habitest Modular System® (Coulbourn Instruments) with a modified opening (⌀ 1.5 cm). The nosepoke contained an infrared (IR) beam and pairs of red, green, and yellow LEDs. The operation of the initiator differed slightly between batches due to temporary technical problems during Batch 1.

- Batch 1: Only the green LEDs were used. The nosepoke was programmed so that by default the green set of LEDs were on signalling that the initiator was available. If the animal broke the IR beam within the nosepoke, all LEDs switched off to signal a correct initiation and that the initiator was briefly deactivated. After 1 s, the green LEDs switched on again to signal that the initiator was available again so the animal could opt for a new trial.
- Batch 2: The green and yellow LEDs were used. The nosepoke was programmed so that by default the both green and yellow sets of LEDs were on signalling that the initiator was available. If the animal broke the IR beam within the nosepoke, all LEDs switched off to signal a correct initiation and that the initiator was briefly deactivated. One second after the IR beam became intact again, i.e. the animal had removed its nose from the nosepoke, the green LEDs switched on to signal that the initiator was available again so the animal could opt for a new trial. The yellow LEDs instead flashed on and off every 250 ms for a further 4 s before remaining on to signal to both the animal and the experimenter the maximum trial length (5s). Any re-break of the IR beam at any point in this process reset the process from the beginning. In this way, if an animal remained with its nose in the nosepoke, or repeatedly nose-poked before the 1 s time-out was up, the initiator would not become available again (signalled by LEDs on and flashing) until the animal had removed its nose for 1s. This ensured the animal would have a chance to focus on the goal-box cues before making a decision to go or re-initiate. Due to the failure of the nosepoke in one apparatus, an improvised nosepoke was set-up in one apparatus using a green LED connected to a breadboard and controlled using an Arduino microcontroller. Whereas no IR beam was present in the nosepoke, the experimenter could trigger the same LED sequence (off for 1 s, flashing for 4 s, then on) with the push of a button if the mouse performed a nosepoke.

Supplementary Table S1. Summary of similarities and differences in design of test arena by species and, where appropriate, by batch.

Design Features		Horses	Rats	Mice*
**Test Arena**	Design**	Bespoke arena (B1: 3.00 x 3.50 m; B2: 5.30 x 2.95 m)	Bespoke apparatus (75 x 50 x 40 cm)	Bespoke apparatus (50 x 25 x 25 cm)
	Goal-box location	Front of arena	Front of arena	Front of arena
	Initiator location	Back of arena	Back of arena	Back of arena
**Goal-box**	Design**	Box (27 x 18 x 14 cm) with hinged lid & perforated floor	Box (6 x 5 x 4 cm) with hinged lid & perforated floor	Hole in apparatus wall (⌀ 1.5 cm) blocked by sliding door
	Operation	Depress lever at back to open lid	Depress lever at back to open lid	Slide door upwards
	Spatial cue	Location of open lid	Location of open lid	Location of open door
	Visual signal	Blue/yellow pattern inside lid	Single white LED under false floor	Single white LED behind hole
	Auditory signal	'Clack' as lid hits wall	'Clack' as lid hits wall	'Clack' as door hits barrier
	Reward delivery	Via hole at back of goal-box	Via delivery chute at back of goal-box	Via delivery chute behind hole
	Odour control	Rewards placed under false floor	Rewards placed under false floor	Reward delivered after choice
**Trial Initiator**	Design	Suspended weighted plastic bottle	Nosepoke (⌀ 2.5 cm) with green and yellow LEDs inside	Nosepoke (⌀ 1.5 cm) with green and yellow LEDs inside[#]
	Correct initiation	Touch bottle with muzzle	Break infrared beam in nosepoke	Break infrared beam in nosepoke
**Signals**	Initiation	Bottle raised (2s), goal-box opens	LEDs switch off (1s), goal-box opens	LEDs switch off (1s), goal-box opens
	Initiator available	Bottle returned to position	LEDs flash	LEDs switch on (B1) or flash (B2)*
	Maximum trial time reached	Goal-box closes	Goal-box closes & LEDs on continuously	Goal-box closes (B1) & LEDs on continuously (B2)[##]

*Combined for C57 & SWISS

** Dimensions are given as l x b or l x b x h.

[#] Except in one apparatus where only green LEDs were present in Batch 2, due to temporary technical problems.

[##] Refer to text for more information.

B1: Batch 1, B2: Batch 2

TRAINING AND TEST PROTOCOL

Summary information per species is provided in Supplementary Table S2 and more detailed descriptions in the following sections.

General information and definition of responses

Horses. Horses were trained and tested in two batches by one experimenter (SH). Animals received one session per day. During habituation, and for part of shaping, the experimenter was present inside the arena, while remaining outside thereafter. The experimenter was then positioned behind the wall with the goal-boxes from where she operated the trial initiator, the goal-boxes, and the delivery of rewards. Rewards were in place in the goal-box when the goal-box lid was opened. Horses were not food restricted but received their regular allowance of feed three times a day.

Go and No-go responses were recorded by the experimenter from the Left-Right Discrimination Stage onwards. A Go response was recorded when the animal's head passed over or touched the open goal-box, while in the Left-Right Discrimination Stage an incorrect Go response was recorded when the animal's head passed over or touched any of the closed goal-boxes first. This was to prevent horses from screening boxes for the presence of reward before taking a decision. A No-go response was recorded if the animal re-initiated or did not make contact with any goal-box within the maximum trial time. From the Go/No-go discrimination stage onwards, only Go responses were recorded as "incorrect" Go responses almost never occured..

Rats. Rats were trained and tested in two batches by one experimenter (SC). Animals received one session per day. Throughout the study, the experimenter was positioned behind the wall with the goal-boxes, from where she operated the goal-boxes and the delivery of rewards. Rewards were in place in the goal-box when the goal-box door was open. Rats were fed a restricted diet of standard rodent food (KLIBA NAFAG #3430, Switzerland) to maintain 90 % of their free-feeding weight.

Go and No-go responses were recorded by the experimenter from the Left-Right Discrimination Stage onwards. Incorrect Go responses were recorded at this stage only. A Go response was recorded when the animal's head or forepaws touched the open goal-box, without touching any other goal-box first, while an incorrect Go response was recorded at the Left-Right Discrimination Stage when the animal's head or forepaws touched any closed goal-box first. This was to prevent the rats from screening the boxes for the presence of reward before taking a decision. As incorrect Go responses almost never occurred after this stage, this response was not recorded in further stages. A No-go response was recorded when the animal re-initiated or did not make contact with any goal-box within the maximum trial time.

Mice. Mice were trained and tested in two batches in three, virtually identical apparatuses by four experimenters in Batch 1 (EM, JDB, MB-S, SH) and three experimenters in Batch 2 (EM, JDB, MB-S). In the majority of cases, individual mice were trained and tested by the same experimenter. However, for mice in Batch 1, one experimenter was replaced (MB-S by SH) towards the end of the training period due to an emergency leave of absence, and for Batch 2, mice from one experimenter (EM) were shaped for two sessions by the other two experimenters (JB and MB-S) due to illness. Up to three mice were tested or trained in the same room simultaneously. Due to the design of the larger experiment (Bailoo, Murphy et al., manuscript in preparation), only one mouse per cage was trained and tested in the JBT. Throughout the study, the experimenters were positioned behind the wall with the goal-boxes, from where they operated the goal-boxes and the delivery of rewards. Rewards were only delivered after the animal had made a Go response. Mice were fed a restricted diet of standard rodent food (KLIBA NAFAG #3430, Switzerland) to maintain 85-90 % (+ 1-3 %) of their free-feeding weight.

Go and No-go responses were recorded by the experimenter from the Left-Right Discrimination stage onwards. A Go response was recorded if the animal placed its nose within the circumference of the goal-box hole. Since the rewards were only delivered after this response was made and due to the design of the apparatus (no physical boxes present), mice were unlikely to investigate closed goal-boxes; incorrect Go responses were thus not recorded. A No-go response was recorded if the animal re-initiated or did not perform a Go response within the maximum trial time.

Training

Stage 1: Habituation

Horses. Horses were used to regular handling prior to this study and no habituation to the reward was necessary, as it was their usual food. Only the positive goal-box was used at this stage. Habituation was deemed successful if the horse stayed calm inside the arena (no whinnying, no fast movements in the arena, no repeated defecation) when the goal-box was opened and closed, and when it readily fed from the positive goal-box.

Rats. Rats were extensively handled in the previous study (Lampe et al., manuscript in preparation), and by the experimenter in the present study before training began. Further, rats were habituated to the rewards in their home cage prior to training. Rats were initially habituated to the test arena in pairs (assigned to the same positive location), and then individually until no obvious signs of fear or anxiety were observed (freezing, defecation, excessive grooming). Only the positive goal-box was used at this stage. Initially the goal-box was open and rewards were freely available within and on the floor to encourage exploration. Once rats were readily eating from the goal-box, the lid was closed after

each reward was obtained and only opened again when the rat was within one body length from the goal-box and facing towards it. Habituation was deemed successful provided rats were consistently moving away and returning to the goal-box when it opened to obtain the reward.

Mice. Mice were extensively handled by three experimenters (EM, JB, MB-S) in their home cage and were habituated to the rewards in their home cage and to the test arena (though without the nosepoke and goal-boxes) prior to this study. Only the positive goal-box was used at this stage. As they were already habituated to the test arena, they were only given one 10-min habituation session in the arena where rewards were initially freely available in the goal-box. Once a mouse had obtained a few of the freely available rewards, rewards were only delivered once the mouse placed its nose within the circumference of the hole in the goal-box. Next, the goal-box door was closed and only opened again when the mouse was close to the goal-box and moving towards it. Mice which failed to eat within this first session were given a second habituation session following the same procedure until all mice were consistently moving away and returning to the goal-box when it opened to obtain the reward.

Stage 2: Shaping for Trial Initiation

Horses. Horses were shaped for trial initiation using the backwards-chaining method (McGreevy and Boakes, 2007), similar to Neave and colleagues (2013). Only the positive goal-box was used at this stage. Horses were trained to first touch the initiator (i.e. the plastic bottle), held by the experimenter to receive a reward (also held by the experimenter). Once a horse had learned to touch the initiator, the initiator was placed in its final position at the back of the arena. The experimenter then gradually moved away from the initiator towards the goal-boxes so that the horse had to move back and forth between the initiator and the experimenter to obtain the reward. From the point where the experimenter reached the goal-boxes, the reward was only provided in the goal-box. Next, the experimenter moved out of the arena and gently tapped the lid of the goal-box against the metal bars of the arena as an auditory signal that the goal-box was open. Horses had to perform 20 full cycles of initiations, i.e. touching the initiator, then moving to the open goal-box and consuming the reward, to move to the next stage.

Rats. Rats were shaped for trial initiation using only the positive goal-box at this stage. Rats were trained to move gradually further from the goal-box and towards the initiator before the experimenter would open the goal-box. The experimenter gently tapped the lid of the goal-box against the wall of the apparatus as an auditory signal that the goal-box was open. Provided the rat consistently returned to obtain the reward in the open goal-box, the experimenter only opened the goal-box as the animal moved increasingly towards the nosepoke, and eventually only when the rat had successfully broken

the IR mean within the nosepoke. Once the reward was consumed, the experimenter gently closed the goal-box lid. Rats had to perform 20 full cycles of initiations within one session, i.e. touching the initiator, then moving to the open goal-box and consuming the reward, to move to the next stage.

Mice. Mice were shaped for trial initiation using only the positive goal-box at this stage. Mice were trained to move gradually further from the goal-box and towards the initiator before the experimenter would open the goal-box. The experimenter gently tapped the door of the goal-box against the barrier as an auditory signal that the goal-box was open. Provided the mouse consistently returned to obtain the reward in the open goal-box, the experimenter only opened the goal box as the animal moved increasingly towards the nosepoke, and eventually only when the mouse had successfully broken the IR beam within the nosepoke. Once the reward was consumed, the experimenter gently closed the goal-box lid. Mice had to perform 20 full cycles of initiations within one session, i.e. touching the initiator, then moving to the open goal-box and consuming the reward, to move to the next stage.

Stage 3: Left-Right Discrimination

Horses. Both the positive and negative goal-boxes were used in this stage and both contained a reward when open. In sessions of 50 trials, where the maximum trial time was 10 s, correct Go responses, incorrect Go responses, and No-go responses were recorded per trial. Horses had to perform 80 % correct Go responses to both positive and negative goal-boxes within a single session to move to the next stage.

Rats. Both the positive and negative goal-boxes were used in this stage and both contained a reward when open. In sessions of 50 trials, where the maximum trial time was 5 s, correct Go responses, incorrect Go responses, and No-go responses were recorded per trial. Rats had to perform 80 % correct Go responses to both positive and negative goal-boxes within a single session to move to the next stage.

Mice. Both the positive and negative goal-boxes were used in this stage and both contained a reward when open. In sessions of 50 trials, where the maximum trial time was 5 s, Go and No-go responses were recorded per trial. Mice had to perform 80 % correct Go responses to both positive and negative goal-boxes within a single session to move to the next stage.

Stage 4: Go/No-go Discrimination

Horses. Both the positive and negative goal-boxes were used in this stage but only the positive goal-box contained a reward when open. In sessions of 50 trials, where the maximum trial time was 10 s, Go and No-go responses were recorded per trial. Sessions were divided into three blocks: Block A (trials 1 - 5 and 26 - 30), Block B (trials 6 - 25), and Block C (trials 31 - 50). Each block consisted of equal

numbers of positive and negative trials. Horses had to perform 80 % Go responses in positive trials and 80 % No-go responses in negative trials in four consecutive blocks of B and C trials to proceed to testing. Due to an experimenter's error, Block A in horses was comprised of different trials compared to rats and mice (see below), however this arrangement was decided *a priori* (i.e. not based on the performance of the horses) and was not deemed to influence the results, considering that the same strict performance criterion was applied across the three species.

Rats. Both the positive and negative goal-boxes were used in this stage but only the positive goal-box contained a reward when open. In sessions of 50 trials, where the maximum trial time was 5 s, Go and No-go responses were recorded per trial. Sessions were divided into three blocks: Block A (trials 1 - 10), Block B (trials 11 - 30), and Block C (trials 31 - 50). Each block consisted of equal numbers of positive and negative trials. Rats had to perform 80 % Go responses in positive trials and 80 % No-go responses in negative trials in four consecutive blocks of B and C trials to proceed to testing.

Mice. Both the positive and negative goal-boxes were used in this stage but only the positive goal-box contained a reward when open. In sessions of 50 trials, where the maximum trial time was 5 s, Go and No-go responses were recorded per trial. Sessions were divided into three blocks: Block A (trials 1 - 10), Block B (trials 11 - 30), and Block C (trials 31 - 50). Each block had equal numbers of positive and negative trials. Mice had to perform 80 % Go responses in positive trials and 80 % No-go responses in negative trials in four consecutive blocks of B and C trials to proceed to testing.

Testing

Stage 5: Judgement Bias Testing

Horses. All goal-boxes were used in this stage; the positive and ambiguous goal-boxes were always rewarded. In six sessions of 53 trials, where the maximum trial time was 10 s, Go and No-go responses were recorded per trial. Ambiguous trials were presented in trials 16, 32, and 48.

Rats. All goal-boxes were used in this stage; the positive and ambiguous goal-boxes were always rewarded. In six sessions of 53 trials, where the maximum trial time was 5 s, Go and No-go responses were recorded per trial. Ambiguous trials were presented in trials 16, 32, and 48.

Mice. All goal-boxes were used in this stage; the positive and ambiguous goal-boxes were always rewarded. In six sessions of 53 trials, where the maximum trial time was 5 s, Go and No-go responses were recorded per trial. Ambiguous trials were presented in trials 16, 32, and 48.

Supplementary Table S2. Summary of similarities and differences in training and test protocol by species and, where appropriate, by batch.

	Horses	Rats	Mice*
**General Set-up**			
Batches	Two	Two	Two
Sessions per day	One	One	One or Two
Experimenters	SH	SC	EM, JB, MB-S, SH**
Interaction with Experimenter	Experimenter present in arena in habituation and shaping stages	No direct interaction during sessions	No direct interaction during sessions
Food restriction	None	To maintain 90 % of their free-feeding weight	To maintain 85-90 % (+1-3%) of their free-feeding weight
Rewards	Rewards same as normal food	Experienced in home cage before starting JBT	Experienced in home cage before starting JBT
Reward delivery	Present in goal-box	Present in goal-box	Triggered by Go response
**Definition of Responses**			
Go response	Animal's head passes over or touches open goal-box	Animal's head/nose or forepaws touch the open goal-box	Animal places nose within the circumference of the goal-box hole
Incorrect Go response	Animal's head passes over or touches any closed goal-box first	Animal's head/nose or forepaws touch any closed goal-box first	N/A as no physical boxes present in arena
No-go response	Animal re-initiates or does not make contact with any goal-box within max. trial time	Animal re-initiates or does not make contact with any goal-box within max. trial time	Animal re-initiates or does not perform Go response within max. trial time

Stages			
**Stage 1**	**Habituation**		
Goal-boxes used	Pos	Pos	Pos
Rewarded goal-box	Pos	Pos	Pos
Handling	Extensively handled due to general use as riding and carriage horses	Handled in home cage before starting JBT	Handled in home cage before starting JBT
Aim	Animal readily consumes reward from goal-box	Animal moves away and returns to goal-box to consume reward	Animal moves away and returns to goal-box to consume reward
**Stage 2**	**Shaping for Trial Initiation**		
Goal-boxes used	Pos	Pos	Pos
Rewarded goal-box	Pos	Pos	Pos
Method used	Shaped to initiate to receive reward first from experimenter, then from goal-box and with experimenter outside arena	Shaped to gradually move further towards initiator to open goal-box	Shaped to gradually move further towards initiator to open goal-box
Aim	20 correct initiations followed by returning to goal-box and consumption of reward		
**Stage 3**	**Left-Right Discrimination**		
Goal-boxes used	Pos, Neg	Pos, Neg	Pos, Neg
Rewarded goal-box	Pos, Neg	Pos, Neg	Pos, Neg
Trials per session	50	50	50
Max. trial time	10 s	5 s	5 s
Aim	80 % correct Go responses to both Pos and Neg goal-boxes within a single session		
**Stage 4**	**Go/No-go Discrimination**		
Goal-boxes used	Pos, Neg	Pos, Neg	Pos, Neg
Rewarded goal-box	Pos	Pos	Pos
Trials per session	50	50	50

Max. trial time	10 s	5 s	5 s
Division of trial blocks	Block A: Trials 1-5 & 26-30<br>Block B: Trials 6-25<br>Block C: Trials 31-50	Block A: Trials 1-10<br>Block B: Trials 11-30<br>Block C: Trials 31-50	Block A: Trials 1-10<br>Block B: Trials 11-30<br>Block C: Trials 31-50
Aim	80% Go responses in positive trials and 80% No-go responses negative trials in four consecutive blocks of B and C trials		
**Stage 5**	**Judgement Bias Test**		
Goal-boxes used	Pos, Neg, NN, M, NP	Pos, Neg, NN, M, NP	Pos, Neg, NN, M, NP
Rewarded goal-box	Pos, NN, M, NP	Pos, NN, M, NP	Pos, NN, M, NP
Trials per session	53	53	53
Max. trial time	10 s	5 s	5 s
Aim	To assess responses to ambiguous cue locations		

*Combined for C57 & SWISS

** Refer to text for more information.
